# A cross-sectional survey on depersonalization/derealization and meditation-induced alterations of the self

**DOI:** 10.1038/s41598-026-51014-y

**Published:** 2026-05-08

**Authors:** Erola Pons, Julieta Galante, Nicholas T. Van Dam, Axel Lindner

**Affiliations:** 1 Department of Psychiatry and Psychotherapy, German Center for Mental Health Tübingen (DZPG), University Hospital of Tübingen, Tübingen, Germany; 2 Contemplative Studies Centre, Melbourne School of Psychological Sciences, University of Melbourne, Melbourne, Australia; 3 Division of Neuropsychology, Center of Neurology, Hertie Institute for Clinical Brain Research, University of Tübingen, Tübingen, Germany

**Keywords:** Meditation, Depersonalization, Derealization, Sense of self, Ego dissolution, Mental health, Diseases, Health care, Medical research, Neuroscience, Psychology, Psychology

## Abstract

Depersonalization/derealization (DPDR) involves feeling detached from one’s body, thoughts, or emotions and is often triggered by trauma, stress, mental health issues, or drug use, typically causing high distress. Similar experiences are also reported in meditation contexts, where they are often described as positive, insightful, and meaningful. However, no study has systematically compared DPDR-like experiences across these contexts. This preregistered, cross-sectional study addresses this gap. Participants who had experienced DPDR-like states triggered through meditation (MEDT, *n* = 60) or through other triggers (NMEDT, *n* = 61) completed five questionnaires and provided additional information about their DPDR experience and meditation practice. The two groups significantly differed on all questionnaires except the Cambridge Depersonalisation Scale, including its subscales covering different DPDR aspects. The MEDT group rated their experiences as substantially more positive than NMEDT, though most participants in both groups described them as mixed. In MEDT, these experiences were reported across meditation types and experience levels. Overall, meditation can induce states phenomenologically similar to DPDR, but these are often experienced as more welcome, pleasant, and spiritually meaningful than those triggered by trauma, stress, or cannabis, although distress is not uncommon. These findings suggest contemplative approaches may inform clinical support for DPDR, while highlighting the need for recognition and guidance within meditation settings.

**Supplementary Information:**

The online version contains supplementary material available at 10.1038/s41598-026-51014-y.

## Introduction

“I would notice my hands and feet moving, but as if they did not belong to me and were moving automatically”^1^. “Music usually moves me, but now it might as well be someone mincing potatoes … I seem to be walking about in a world I recognise but don’t feel”^1^.

These quotes are from individuals suffering from depersonalization/derealization (DPDR) disorder, a condition marked by a pervasive sense of disconnection either from oneself or the external world, or both. Depersonalization (DP) involves feeling detached from one’s own thoughts, emotions, or body, like observing oneself from the outside, as if being a robot or losing the sense of being a unified, embodied self. Derealization (DR), by contrast, refers to a sense of detachment from one’s surroundings, which may appear unreal, foggy, dreamlike, or visually distorted^2^. People with DPDR often report feeling emotionally numb, like passive observers of their own lives. The condition is typically triggered by traumatic or highly stressful events, cannabis use, or neurological conditions such as stroke or epilepsy, and is usually experienced as uncontrollable, distressing, and impairing^2^.

“I was talking to someone and all of a sudden it felt like I wasn’t doing the talking”^3^. “It was like being a pure impersonal observer watching a movie … There was a real pureness and coolness, and noninvolvement, and no emotions”^3^. “A certain calmness pervaded everything”^3^.

These reports come from advanced meditators describing states commonly associated with meditation. In many Buddhist traditions, the self is seen as an illusion, a mental construct made of impermanent elements like body, thoughts, and emotions. Meditation is thought to reveal this illusion by helping practitioners observe their mental processes more clearly, leading to disidentification from the sense of a separate, enduring self^4,5^. This disidentification may give rise to states that resemble DPDR but are typically positively valenced when they arise intentionally and are seen as meaningful insights. However, the same states may feel confusing or disturbing, similarly to DPDR patients^6^.

DPDR-like experiences can therefore be appraised in radically different ways: as meaningful and beneficial when intentionally cultivated through meditation, or as distressing and disorienting when arising unexpectedly. Despite this paradox, the topic has received limited scientific attention. Most research consists of case studies or qualitative work with experienced meditators, reporting both positive^3,7–11^ and distressing^9,12–17^ experiences. Some theoretical efforts have linked meditation-related dissolution of self to DPDR^18–20^, and one qualitative study^13^ interviewed both meditators with negative experiences and clinical patients. Yet, no quantitative study has directly compared these groups, and the current study aims to fill this gap.

In this preregistered cross-sectional study, we investigated how DPDR-like experiences differ depending on the context in which they arise, specifically, whether they are triggered through meditation (MEDT group) or through other means (NMEDT group). Our primary aim was to examine whether the overall phenomenology of the experiences was comparable across groups, which we assessed using the Cambridge depersonalisation scale (CDS)^21^ and its five-factor structure. We hypothesized that the experiences would be broadly similar. We then explored group differences in emotional tone and meaning. Specifically, we compared the groups in terms of the emotional valence attributed to the experiences (pleasantness vs. unpleasantness) as well as the presence of difficult emotions and sensations via the challenging experiences questionnaire (CEQ)^22^, expecting greater negativity in the NMEDT group. We also examined the perceived spiritual significance of the experiences via the mysticism scale (MS)^23^ and ego dissolution inventory (EDI)^24^, expecting higher scores in the MEDT group. Finally, we compared five facet mindfulness questionnaire (FFMQ)^25^ scores between groups to explore whether differences in experience appraisal might reflect trait mindfulness, where we also expected higher scores in the MEDT group. We also discuss the implications of this study, ranging from clinical relevance to addressing foundational philosophical questions about the sense of self.

## Results

### Group characteristics

All assessed demographic and clinical characteristics (Table 1) differed significantly between groups: the NMEDT group had a higher proportion of females, was younger, reported fewer lifetime hours of meditation practice, and had a higher prevalence of self-reported current mental disorders. The majority of participants in both groups (> 90%) lived in a Western country (Europe, North America, or Australia and New Zealand).

**Table 1:** Demographic and clinical data for the meditation and non-meditation trigger groups.

	Meditation trigger MEDT (*n* = 60)	Non-meditation trigger NMEDT (*n* = 61)	*p*-value
**Gender**			< 0.001
Female	15 (25%)	35 (57.4%)	
Male	43 (71.7%)	21 (34.4%)	
Diverse	2 (3.3%)	5 (8.2%)	
**Age**	33 [26–39.2]	26 [22–31]	< 0.001
**Lifetime Meditation (hours)**	1114 [495–3592]	5 [0–40]	< 0.001
**Current mental disorders (any)**	7 (11.7%)	26 (42.6%)	< 0.001
Depression	6 (10.0%)	16 (26.2%)	
Anxiety disorders	2 (3.3%)	13 (21.3%)	
Other	1 (1.7%)	12 (19.7%)	

Numbers are presented as counts and percentage (%) or median and interquartile range [IQR], as appropriate. Chi-square tests were used for Gender (Diverse was excluded from this comparison due to the low *n*) and Mental Disorders (any). Permutation tests were applied to Age and Hours of Meditation (*n* = 52 for the meditation group). Note that Mental Disorders (any) does not necessarily result from summing up the options listed below it, as several participants self-reported more than one diagnosis.

Of all participants, only four had received an official DPDR diagnosis from a psychiatrist, one following an episode triggered by meditation and the rest due to other triggers. However, on the Cambridge depersonalisation scale (CDS), most participants in both groups scored above 70, the clinical cutoff for DPDR^21^, 61.7% in MEDT and 77.0% in NMEDT (permutation test, *p* = 0.048).

In the NMEDT group, the primary triggers of DPDR experiences were stress/anxiety (*n* = 45, 73.8%), trauma (*n* = 22, 36.1%), depression (*n* = 18, 29.5%), cannabis (DPDR lasting longer than the acute effect of the drug; *n* = 6, 9.8%), and other (*n* = 21, 34.4%). The total percentage exceeds 100% because many participants experienced DPDR due to multiple triggers. Within the “other” category, participants reported various triggers, including spontaneous occurrences in daily life (*n* = 3), OCD (*n* = 2), pain (*n* = 2), surgery (*n* = 1), and psychedelics (DPDR persisting beyond the drug’s acute pharmacological effects; *n* = 1).

### Questionnaire scores

**Fig. 1:**
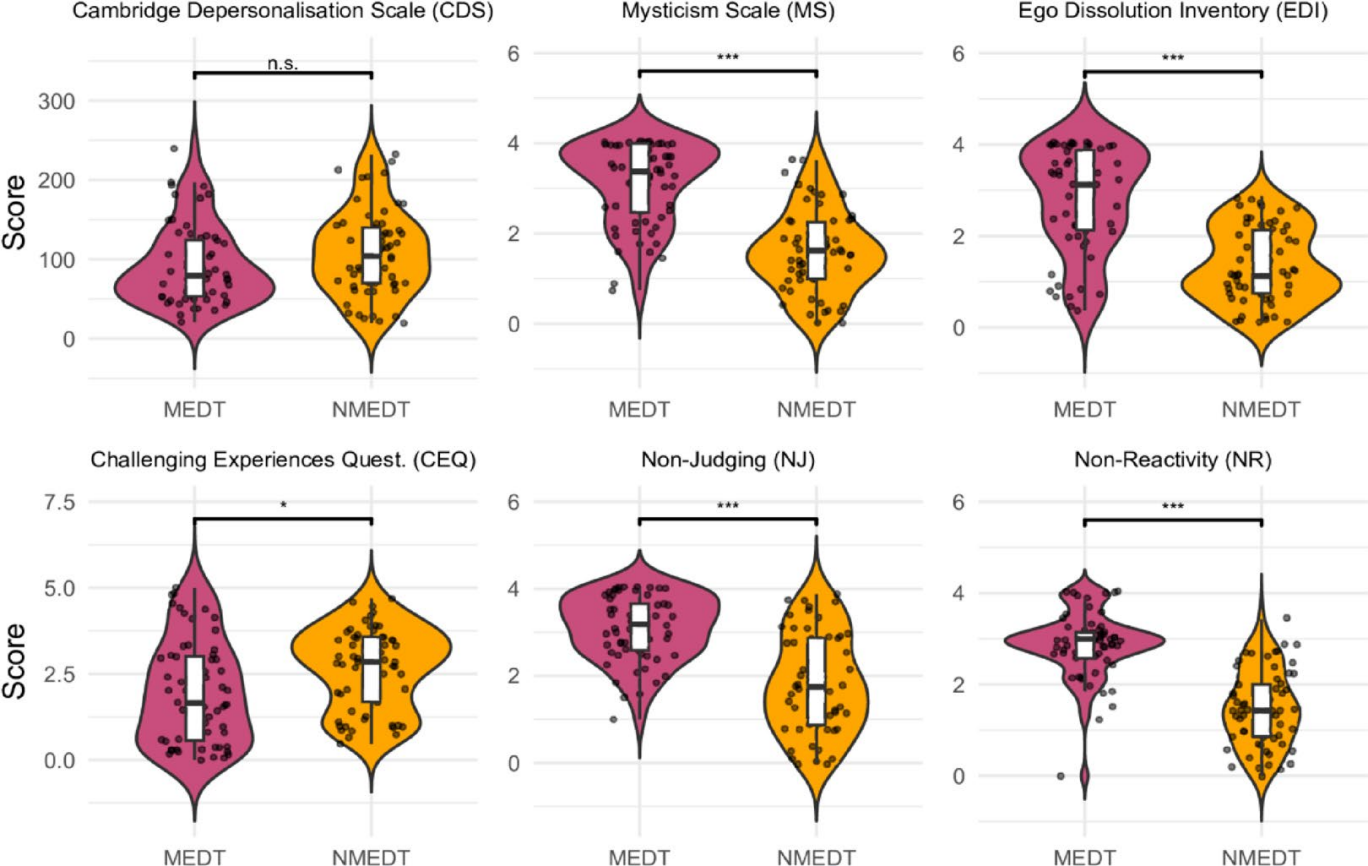
Questionnaire scores for the meditation (MEDT) and non-meditation (NMEDT) trigger groups. Violin shapes represent the distribution of individual scores; embedded boxplots show the median (line), interquartile range (box), and range excluding outliers (whiskers). Individual dots represent participants. Significance symbols (* < 0.05, ** < 0.01, *** < 0.001) reflect Bonferroni-corrected comparisons.

A MANCOVA revealed a significant multivariate effect of Trigger (MEDT vs. NMEDT) on the combined questionnaire scores, after controlling for gender, age, and mental health diagnosis: *Pillai’s Trace* = 0.644, *F*(6, 111) = 33.46, *p* < 0.001, *η*_p_^2^ = 0.39. Neither gender, age, nor mental health diagnosis had a significant multivariate effect, *p* > 0.05. Due to violations of parametric assumptions, follow-up analyses were conducted using permutation tests for each of the six questionnaire measures, with effect sizes calculated using standard parametric formulas (Fig. 1, Table S1). There were no significant differences in CDS scores between the two groups. However, as expected, the MEDT group scored significantly higher, with large effect sizes, on the Mysticism Scale (MS), Ego Dissolution Inventory (EDI), and the two facets of Five Facet Mindfulness Questionnaire that were used, Non-Judging (NJ) and Non-Reactivity (NR) to inner experience (*p*_corr._ < 0.001). Meanwhile, the NMEDT group scored higher on the Challenging Experiences Questionnaire (CEQ) (*p*_corr._ = 0.044), though the effect size was substantially smaller. Excluding one identified outlier on the NR questionnaire within the MEDT group did not substantially affect the results.

We repeated the analysis including only participants scoring ≥ 70 on the CDS (the clinical cutoff for DPDR; MEDT: *n* = 37, 61.7%; NMEDT: *n* = 47, 77.9%). Demographic and clinical profiles closely resembled those of the full sample (e.g., current mental disorder: 10.8% in MEDT vs. 44.7% in NMEDT). The respective MANCOVA and permutation tests remained largely unchanged, except that the group difference on the CEQ was no longer significant (*p*_*corr.*_ = 0.564) (Supplementary Table S2), suggesting that strong DPDR-like experiences triggered by meditation can also be quite unpleasant.

Importantly, within-subject permutation tests on MEDT participants completing surveys also for other triggers (*n* = 8) supported that score differences were due to triggers: CDS non-significant; MS *p* = 0.032; EDI *p* = 0.038; CEQ *p* = 0.007; NJ *p* = 0.016; NR *p* = 0.079.

To examine the potential impact of gender imbalance, we repeated the analyses using repeated gender-balanced subsampling (5,000 iterations; MEDT: 15F, 15 M; NMEDT: 21F, 21 M). Effect sizes were comparable to the original analysis, and effects for MS, EDI, NJ, and NR were significant in at least 99.9% of iterations after correction, for CEQ, 24.8%, and for CDS, 0.3%.

### Emotional valence

**Fig. 2:**
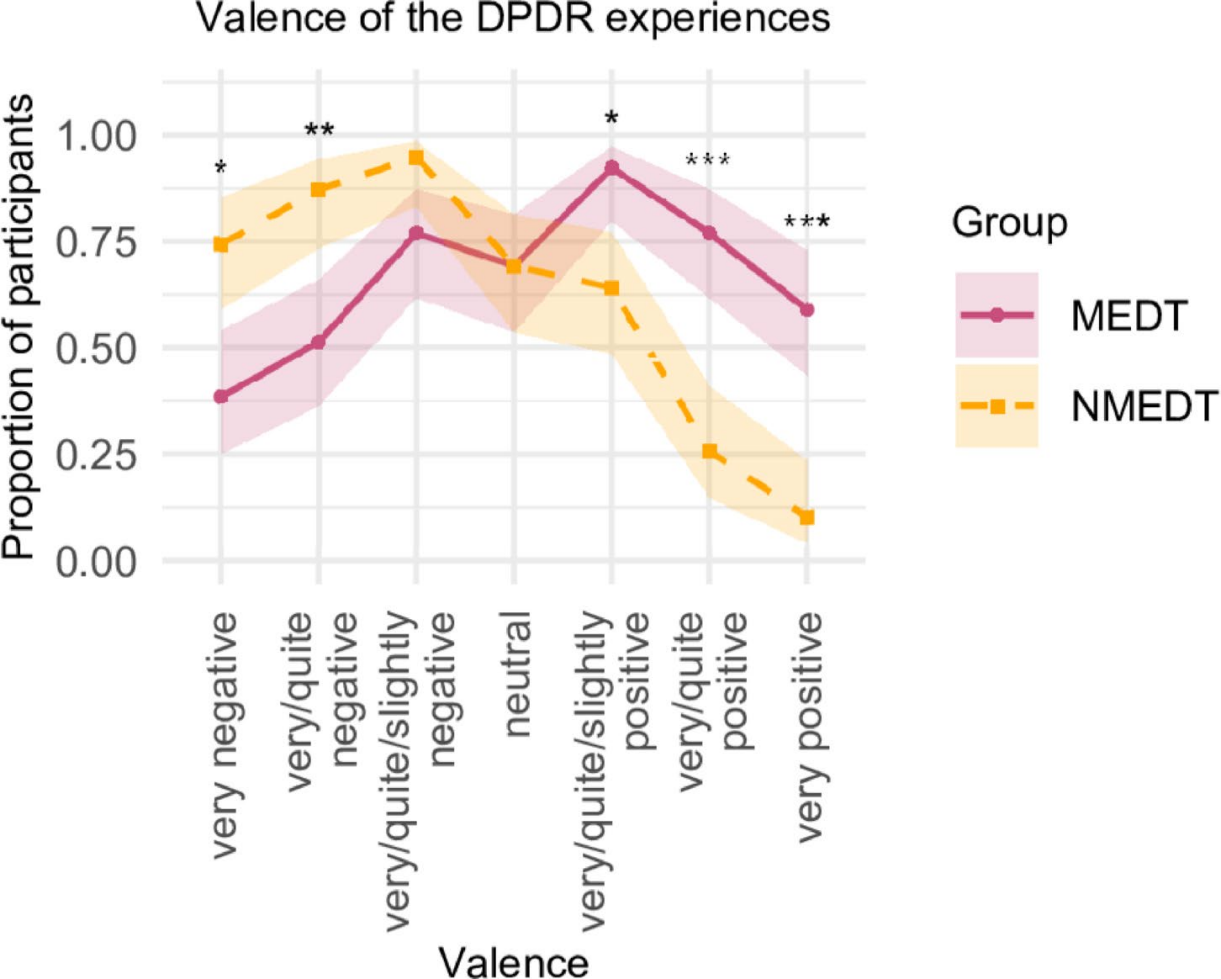
Proportions of participants in each group selecting different emotional valences for their DPDR experiences. Shaded areas represent 95% CIs. Significance symbols (* < 0.05, ** < 0.01, *** < 0.001) reflect Bonferroni-corrected chi-square test comparisons. Note that raw proportions are only shown for ‘very negative’, ‘neutral’, and ‘very positive’; the remaining categories reflect cumulative proportions (e.g., ‘very/quite negative’ = proportion of participants who selected either ‘very negative’ or ‘quite negative’). See Supplementary Fig. S2 for the graph of raw proportions and analyses limited to participants with a CDS score ≥ 70.

Participants rated the emotional valence of their DPDR-like states on a 7-point scale (1 = Very Negative/Unpleasant to 7 = Very Positive/Pleasant), selecting all that applied (i.e. they could, e.g., report positive AND negative valences). This allowed for the possibility that experiences could be perceived as negative, positive, or both within or across episodes. The average number of valences endorsed did not differ significantly between groups (MEDT = 4.18; NMEDT = 3.77; *p* > 0.05) (see Supplementary Fig. S1). Mean valence scores were first averaged within participants and then within each group. On average, the MEDT group reported a more positive valence (*M* = 4.38, *SD* = 0.98, *n* = 39) than the NMEDT group (*M* = 3.01, *SD* = 0.98, *n* = 39), *p* < 0.001, Cohen’s *d* = 1.40. As can be seen in Fig. 2, a larger proportion of participants in the MEDT group selected “Slightly positive/pleasant”, “Quite positive/pleasant”, or “Very positive/pleasant” (92%) compared to the NMEDT group (64%) (*p*_corr._ < 0.01). Conversely, a higher proportion of NMEDT participants endorsed “Slightly negative/unpleasant”, “Quite negative/unpleasant”, or “Very negative/unpleasant” (95%) compared to the MEDT group (77%), although this difference was not statistically significant after Bonferroni correction. More participants in the MEDT group selected “Very positive/pleasant” (*p*_corr._ < 0.001), while more in the NMEDT group selected “Very negative/unpleasant” (*p*_corr._ < 0.05). Neutral experiences were reported by 69% of participants in both groups.

Participants also reported the emotional valence for each individual item of the CDS, MS, and EDI questionnaires. Comparisons across the groups revealed significant differences: as expected, the MEDT group consistently reported higher (i.e. more positive) valence ratings (*p*_corr._ < 0.001) (Supplementary Table S3).

### Phenomenology

Among the 46 MEDT participants who responded to the question of whether their experiences resembled depersonalization (DP), derealization (DR), or both, 37 (80.4%) reported both, 7 (15.2%) reported only DP, and 2 (4.3%) reported only DR. In the NMEDT group, 42 participants provided a response, with 33 (71.7%) reporting both, 3 (6.5%) reporting DP, and 6 (13.0%) reporting DR.

Regarding the five factors of the CDS previously identified^26^, Unreality of Surroundings and Temporal Disintegration showed significant group differences, although these did not survive Bonferroni correction, while Numbing, Unreality of Self, and Perceptual Alterations showed no significant differences (Supplementary Table S4).

Furthermore, two of the five most endorsed items were the same across both groups, while the remaining items differed (Supplementary Table S5).

### Correlation analyses

**Fig. 3:**
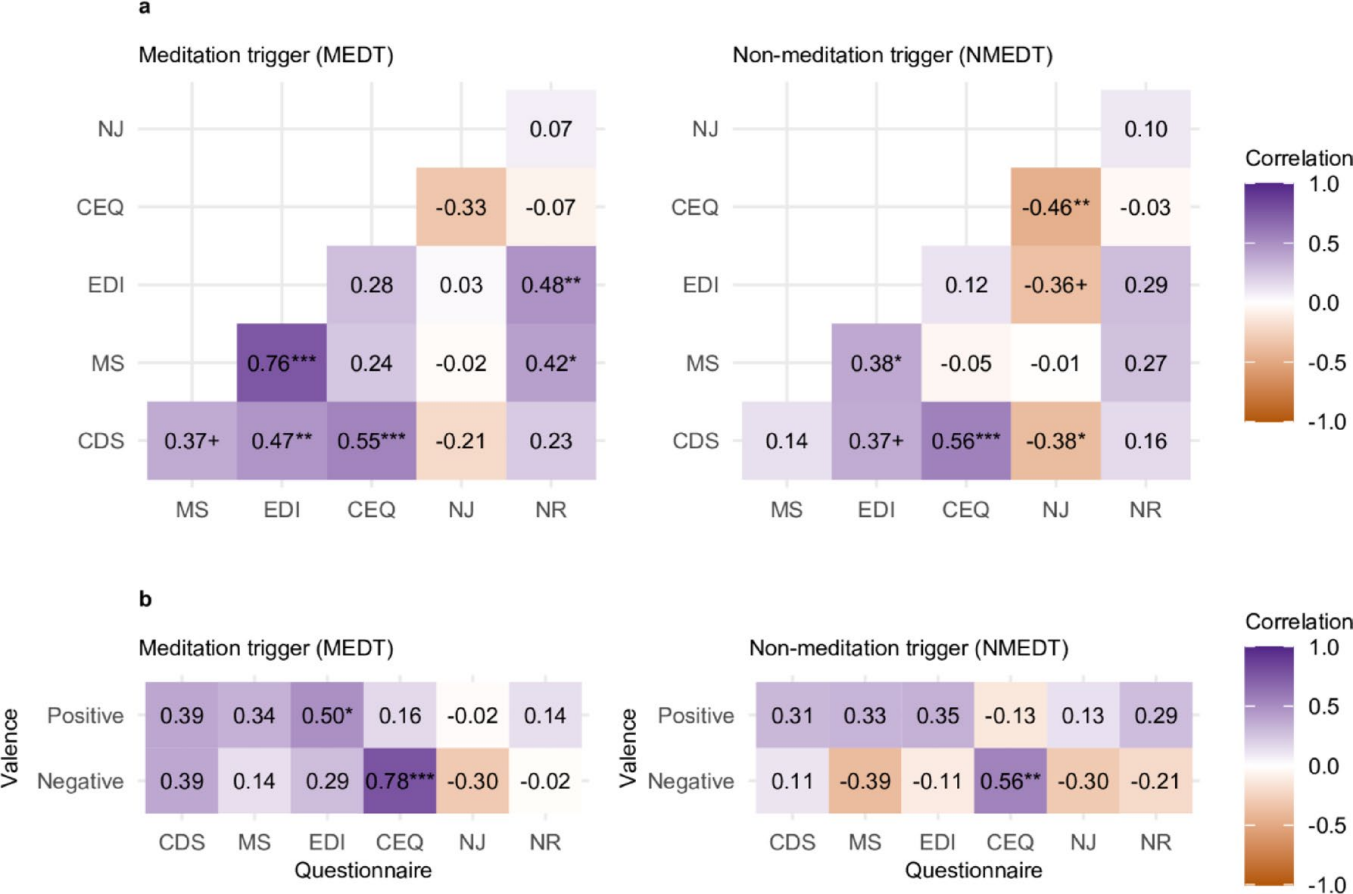
Correlation matrices for questionnaire scores (**A**) and correlations between triggers and questionnaire scores (**B**). Questionnaires: Cambridge Depersonalization Scale (CDS), Mysticism Scale (MS), Ego Dissolution Inventory (EDI), Challenging Experience Questionnaire (CEQ), Non-Judging (NJ), and Non-Reactivity (NR). Significance symbols (+ < 0.1, * < 0.05, ** < 0.01, *** < 0.001) reflect Bonferroni-corrected p-values. In B, valence scores are derived from participants’ selections on a 7-point scale in which they could check all levels of valence that applied to their DPDR experience, ranging from “very negative” to “very positive.” For the positive valence score, responses were coded as 3 if “very positive” was selected (regardless of whether other positive valences were selected), 2 if “somewhat positive,” 1 if “slightly positive,” and 0 if no positive valence was endorsed. The same procedure was applied to compute the negative valence score, coding 3 for “very negative,” 2 for “somewhat negative,” 1 for “slightly negative,” and 0 if no negative valence was selected.

Separate planned correlation analyses for each group revealed several notable associations between the questionnaire scores (Fig. 3A). Overall, CDS, MS, EDI, and CEQ exhibited positive correlations in most pairwise comparisons, particularly in the MEDT group. In this group, NR also correlated positively with several other questionnaires, whereas in the NMEDT group, NJ showed negative correlations with multiple measures.

Additionally, CEQ scores correlated strongly with negative valence of the DPDR experiences in both groups, while EDI scores correlated with positive valence only in MEDT (Fig. 3B).

### Additional findings within the MEDT group

**Fig. 4:**
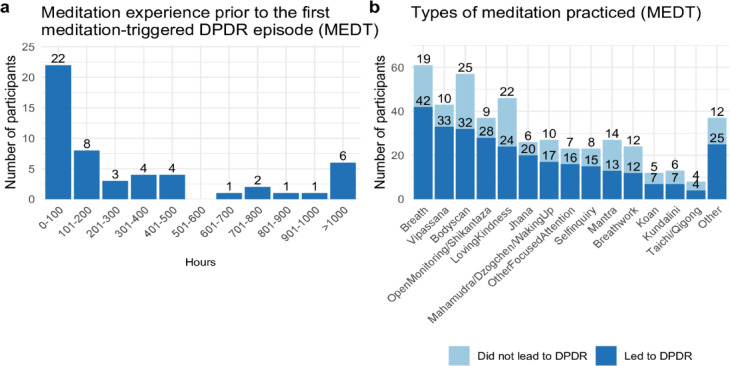
Meditation experience in the MEDT group. (**A**) Histogram of hours of meditation practice prior to the first DPDR-like episode (*n* = 52). (**B**) Histogram of meditation types practiced by participants (*n* = 46) that have triggered DPDR-like experiences at least once vs. those that did not.

For the MEDT group, the number of meditation hours *prior* to the first DPDR experience varied widely and was non-normally distributed, ranging from 5 to 12,200 h, with a median of 175 h (*n* = 52, as some participants did not provide this information) (Fig. 4A). Interestingly, 22 participants (36.5%) reported DPDR-like episodes after relatively little meditation (≤ 100 h), and 32 participants (61.5%) reported not having attended a meditation retreat before the first episode. However, in follow-up analyses, neither hours of meditation practice prior to the first DPDR-like experience nor lifetime meditation hours were associated with positive valence, negative valence, or questionnaire scores (all *p*_corr._ > 0.05).

Participants were presented with a list of meditation types (e.g., focus on the breath, Vipassana, body scan, Shikantaza, loving-kindness) and asked to indicate which they had practiced and whether any had triggered DPDR-like experiences. Across responses, every listed type had been identified by at least some participants as having, at some point, triggered a DPDR-like state, and most participants reported multiple forms of meditation as having triggered it at some point (Fig. 4B). Participants who selected “Other” were asked to describe their practice in an open-text field (see Supplementary Table S6).

We ran two follow-up analyses within the MEDT group: one comparing participants who had experienced DPDR-like states also due to psychedelics (25 participants, 41.7%) vs. those who had not, and another comparing those who had experienced such states also due to other triggers (e.g., cannabis, depression, stress, anxiety, trauma; 30 participants, 50.0%) vs. those who had not. In each analysis, we ran a permutation test per questionnaire, for positive valence and for negative valence; none of the 16 tests reached significance after Bonferroni correction (all *p*_corr._ > 0.05) and only one test was significant before correction (NJ for the comparison on ‘other triggers’: *p* = 0.036) (Supplementary Tables S7 and S8), suggesting these additional triggers did not confound the main group comparisons.

The MEDT group additionally indicated, for each of the 29 CDS items, when these experiences occurred in relation to their meditation sessions. Across participants (*n* = 44), of the 768 of items that were endorsed (60.2%), 12.0% were reported as occurring only during meditation, 13.3% only outside of meditation, and 23.7% as experiences that can occur both during and outside of meditation. Additionally, 20.3% of endorsed items were said to linger for minutes or hours post-meditation, 14.8% for days or weeks post-meditation, and 15.9% were described as a permanent shift. The most frequently endorsed items in the ‘permanent shift’ category were CDS-26 (“I feel/felt so detached from my thoughts that they seem/seemed to have a ‘life’ of their own.”) (*n* = 7) and CDS-16 (“I feel/felt detached from memories of things that have/had happened to me—as if I had not been involved in them.”) (*n* = 6). A total of 25 participants (57%) reported that at least one item was a permanent shift.

## Discussion

Our preregistered study compared individuals whose DPDR-like experiences arose during meditation (MEDT group) to those whose experiences were triggered by other factors (e.g., trauma, anxiety, cannabis; NMEDT group). Using self-report questionnaires, we found several key similarities and differences between the groups.

Cambridge Depersonalisation Scale (CDS) total and subscale scores did not differ significantly between groups, supporting the idea that meditation can elicit DPDR-like experiences. This had been previously discussed from a theoretical perspective^18,19^ and, more prominently, through case reports and qualitative research. Much of this work focuses on advanced meditators who describe alterations in the sense of self that resemble DPDR symptoms, sometimes as positive, insightful experiences^3,7–11^, and other times as disruptive and distressing^9,12,13,13–17^.

CDS scores in both groups (MEDT: 92.8, NMEDT: 108.5) were slightly lower but still comparable to those in clinical DPDR populations (typically around 110–120^21,26,27^).

Over 60% in MEDT and nearly 80% in NMEDT scored above the clinical cutoff of 70, with 75–79% of DPDR patients scoring above this threshold^21,26^. However, only four people had an official diagnosis. Importantly, not all individuals scoring above the cutoff would meet diagnostic criteria, particularly in the MEDT group, where DPDR-like states are often experienced as positive and do not necessarily involve functional impairment. Diagnostic clarity is relevant primarily when symptoms cause clinically significant distress or impairment. However, given that a substantial proportion of individuals with DPDR do report significant negative emotions associated with the experience, the gap between official rates (0.007%) and estimated prevalence of 1–2%^28^ suggests that many qualifying cases may remain undiagnosed. In such instances, recognition is important for validating suffering and facilitating therapy.

The groups diverged strongly in how their DPDR-like states were experienced. The MEDT group scored higher on the mysticism scale (MS) and ego dissolution inventory (EDI), suggesting these states were often perceived as spiritual or transcendent.

Notably, EDI scores in our sample were high, comparable to those reported in psychedelic studies^24^, with the MEDT group exceeding the levels observed in those prior studies.

CDS and EDI scores correlated significantly only in the MEDT group, suggesting overlap between DPDR and ego dissolution in meditative contexts. This contrasts with Sleight et al.^29^, who found a strong link between the dissociative experiences scale (DES-II) and the ego dissolution scale (EDS), specifically with the ego-loss subscale, not unity. The EDI does not separate these components, which may explain the discrepancy. Still, we replicated their ego dissolution–MS correlation in MEDT, reinforcing links between ego dissolution and spiritual experiences.

Participants in the MEDT group were more likely to rate their experience as positive and less likely to rate it as negative than those in the NMEDT group. Furthermore, the NMEDT group scored higher on the challenging experiences questionnaire (CEQ), consistent with the literature describing DPDR as distressing^1,30^. Most of the NMEDT triggers (e.g., stress/anxiety, trauma, depression) are inherently painful, which may have a strong influence of DPDR being unpleasant.

However, in the MEDT group, emotional valence as well as CEQ scores varied widely: while many reported low distress, others scored high, consistent with prior reports that meditation-induced alterations in self and reality span a broad valence range^31^ and can also lead to psychological struggle ^9^.

When analyzing only high-CDS scorers (≥ 70), the CEQ difference between groups disappeared. This may suggest that distress increases with DPDR intensity in meditators. Accordingly, the CDS was strongly positively correlated with the CEQ in both groups. Notably, the more similar CEQ scores among high-CDS participants did not result from more similar rates of mental health issues, as these rates remained nearly unchanged compared to the full sample.

On both non-judging (nj) and non-reactivity (NR) subscales of the FFMQ, the MEDT group scored higher. These traits might help explain their less negative appraisal of DPDR: they may have been more accepting of strange internal states. Supporting this, NR was positively correlated with EDI and MS in the MEDT group, while in NMEDT, NJ was negatively correlated with both CDS and CEQ.

Over a third of MEDT participants reported their first DPDR-like episode after < 100 h of practice, and most had never attended a retreat. This is consistent with previous findings showing that even brief meditation interventions, such as an 8-week course, can elicit altered states of consciousness^32^ and challenges the idea that only expert practitioners enter these states. These findings highlight the importance of greater awareness of such states and of providing appropriate interpretative frameworks within meditation programs, including MBSR or meditation apps. Clear contextualization may facilitate the integration of such experiences in growth-promoting and beneficial ways, while also reducing the likelihood that they are perceived as confusing or distressing.

Timing was highly varied: some states emerged during meditation, others after, and some became ongoing, underscoring meditation’s power to reshape the sense of self in long-lasting ways. This pattern echoes earlier reports that “no-self” or depersonalization-like experiences can arise at different stages of meditation practice and, in some cases, persist beyond formal practice into daily life^9^.

### Limitations

Our study has several limitations. The two groups differed on various demographic and clinical variables (some statistically controlled), and likely also on less measurable traits, like personality, beliefs, or spiritual exposure, that may have shaped both the nature and interpretation of their experiences. For instance, the gender imbalance between groups may have affected valence ratings. As women generally report both higher positive and negative affect^33^, this could have attenuated group differences in positive valence while amplifying differences in negative valence, but potentially balancing out in the overall average. The trigger types were also heterogeneous: while any form of meditation qualified in the MEDT group, the NMEDT group combined very different triggers (e.g., trauma, anxiety, cannabis, pain), which likely differ in mechanisms and impact. However, follow-up analyses, including within-subject comparisons (for participants who completed surveys for both triggers), subgroup tests within the MEDT group, and repeated gender-balanced resampling analyses, suggested that the core differences were indeed due to the triggers.

Another limitation is the adaptation of questionnaire instructions and response formats, which may have affected the internal validity and comparability of the scales. This applies especially to the CDS. For instance, to rate symptom frequency, participants were instructed to consider the period between the first and last DPDR episode, but some might have interpreted it as relative to the episodes.

Also, despite similar CDS scores across groups, we cannot conclude the phenomenology was identical: language may not fully capture these states. Some MEDT participants felt items like “*I felt so detached from my thoughts that they seemed to have a ‘life’ of their own*” or “*When I moved, it didn’t feel like I was in charge of the movements … as if I were a robot*” only partially applied, resonating with the core idea, but not with the pathological framing (‘detached’ or ‘robot’).

Such negatively toned items in the CDS may partly explain its correlation with the CEQ. However, while CEQ scores were linked to more negative overall valence, CDS showed little to no such correlation, highlighting an apparent inconsistency that requires further investigation.

Furthermore, we did not assess contextual aspects of participants’ meditation practice (e.g., whether it occurred within a structured interpretative framework such as Buddhism), beyond hours and type. Accordingly, we cannot determine how such contexts may have shaped the emotional valence or spiritual interpretations of the reported DPDR-like experiences triggered by meditation.

Additionally, approximately half of the MEDT group reported having experienced DPDR-like states triggered by factors other than meditation. Although follow-up analyses did not indicate that this was associated with differences in questionnaire scores or valence ratings, we cannot exclude the possibility that prior or concurrent non-meditation-triggered experiences shaped participants’ interpretations of their meditation-triggered states. Moreover, we did not assess the temporal order of these experiences, limiting our ability to determine potential directional influences.

In the MEDT group, meditation experience ranged widely (5–12,200 h). Although follow-up analyses did not reveal associations between hours of practice (prior to the first DPDR-like experience or lifetime) and questionnaire scores or valence ratings, it remains plausible that greater cumulative engagement may influence the types of altered states encountered and the interpretative frameworks applied to them, adding heterogeneity to our sample.

Finally, some participants in the NMEDT group had prior meditation experience, which may have shifted their interpretation of DPDR-like experiences toward that of the MEDT group, potentially attenuating observed between-group differences.

### Relevance and future directions

This study has several implications, including clinical and theoretical. Despite affecting an estimated 1–2% of the population^34^, DPDR remains poorly understood, with limited research on its mechanisms or treatment. Exploring positively-valenced DPDR, especially through future qualitative work, could help reframe distressing symptoms, suggest new coping strategies, and even open pathways for volitional control over these states, potentially through meditation itself^35–37^.

Since no-self states can be experienced as insightful, meaningful, and growth-promoting in a meditation context, an open empirical question for future research is whether similar states arising through other triggers might acquire comparable benefits through reframing and integration. An opposing hypothesis is that negatively valenced DPDR states primarily function as defensive responses to destabilization or psychopathology, such that attempts to further dissolve the sense of self may be premature; in such cases, working on promoting a healthier sense of self might be a necessary precondition for safe integration.

Although meditation is widely practiced, for instance, by 18% of adults in the US^38^ and 33% in Australia^39^, who report meditating at least once within the past 12 months, potential alterations in the sense of self, remain largely unacknowledged. This is especially true in secular contexts like MBSR programs or meditation apps, where such phenomena are rarely addressed. As a result, practitioners, particularly those without a teacher or guided by someone unfamiliar with these states, may be unprepared if they arise. Even when teachers are aware, they may not consistently prepare students. Importantly, alterations in the sense of self should not be equated with pathology by default. As evidenced in psychedelic therapy research, comparable phenomena can be integrated constructively when embedded within an appropriate interpretative and supportive framework. In our sample, DPDR-like experiences occurred across a wide range of meditative practices and in individuals with varying levels of experience, including those with very few lifetime hours, suggesting these reactions are not limited to advanced or intensive practice. These patterns underscore the need for better recognition of these experiences and their integration into teacher training, apps, and public-facing materials.

Under certain conditions, shifts in self-experience can be beneficial. Reduced identification with thoughts and self-related narratives (decentering) is an effective strategy for reducing depression and anxiety, by weakening the impact of negative inner events^40,41^. In parallel, shifting the sense of self from a fixed, defensive entity to one experienced as interconnected and less attached, for instance, through mindfulness interventions, may promote prosocial behavior^42^. This raises the possibility that shifts resembling DPDR could, with the appropriate framework, promote wellbeing and prosociality.

Our research also bears on foundational psychological and philosophical questions about agency, embodiment, and the construction of selfhood. For instance, one can situate the sense of self within a Bayesian or predictive processing framework, conceptualizing it as a hierarchical model that integrates priors about agency, ownership, and self–other boundaries^43,44^. Empirical work could then compare individuals experiencing DPDR-like states across different triggers and valences to test whether such states are associated with systematic shifts in the precision weighting of these priors, using behavioral and/or neuroimaging approaches.

Future research could directly compare the neural correlates of DPDR and meditation-induced no-self states. Some overlap may be expected in regions involved in body representation, such as the temporoparietal junction^45^, as well as in limbic structures such as the amygdala, whose reactivity to emotional stimuli has been found decreased both in DPDR^46,47^ and meditators^48^. However, the underlying dynamics of such alterations likely diverge. In DPDR, the amygdala hypoactivation is accompanied by prefrontal hyperactivation (frontolimbic overmodulation), and the anterior insula response to emotional stimulation is reduced^49,50^, whereas it is increased in meditation, consistent with preserved or enhanced interoceptive awareness^51,52^. Additionally, the Default Mode Network has been found altered in both populations^53–55^, but in different ways, potentially reflecting heightened maladaptive self-referential processing in DPDR and reduced narrative self-focus in meditation.

## Conclusions

DPDR-like experiences can arise in a wide range of contexts, from meditation to trauma, stress, or drug use, and while their core phenomenology appears consistent, the emotional tone and meaning differ. When triggered by non-contemplative contexts, these experiences tend to be confusing and distressing. In contrast, when arising through meditation, they are often seen as positive and spiritually meaningful, though they can also be deeply unsettling. A deeper awareness and understanding of these nuances is needed, and contemplative frameworks may offer valuable tools for supporting those struggling with similar experiences in different contexts.

## Methods

The study was approved by the Ethics Committee of the University Clinic of the University of Tuebingen. All methods were carried out according to the principles of the Declaration of Helsinki. All participants gave written informed consent prior to participation in the study and were compensated at a rate of €10 per hour (or equivalent). The procedures of this study have been preregistered on OSF (https://doi.org/10.17605/OSF.IO/Y3J59) with partially existing data before any human observation.

### Participants and data collection

We recruited participants who reported experiences consistent with depersonalization (“feeling detached from oneself, as if observing one’s body, actions, or thoughts from the outside, lacking emotional connection, or experiencing the self as unreal or absent”) and/or derealization (“feeling detached from one’s surroundings, with people or objects appearing strange, dreamlike, lifeless, or distorted”). The invitation clarified that these experiences could be either positively or negatively valenced and could stem from a range of triggers (e.g., meditation, trauma, stress, depression). Eligible participants were 18 years or older, with or without a formal diagnosis of Depersonalization/Derealization Disorder and with or without meditation experience. Individuals whose DPDR experiences occurred solely during the acute effects of psychedelics, cannabis, or in the context of psychosis were excluded.

According to the preregistered power analysis (G*Power; α = 0.05, power = 0.90) for a between-subjects MANCOVA with an expected medium effect size of f2(V) = 0.15, for two groups and six scores of interest, we required a total sample of 117 participants. We aimed for balanced groups with nearly equal sizes and recruited 121 participants in total (N_MEDT_ = 60; N_NMEDT_ = 61).

Participants were recruited online via the university-wide email distribution list of the University of Tuebingen (Germany), newsletters of meditation communities, and online meditation forums (such as Discord and Reddit). Upon submitting a brief description of their experiences, the first author assessed whether they aligned with the aforementioned descriptions of DPDR. The study was conducted online via video call with the first author, during which participants completed the survey on their own computer while on the call, allowing them to ask any questions if needed. If participants had experienced DPDR states in multiple contexts and one of those was meditation, they were instructed to complete the survey based on the meditation context, as such cases were more difficult to recruit. Some of these participants also completed a second survey based on a non-meditation trigger, which was not used in the main analysis but included in an exploratory within-subjects comparison. Participants without meditation-related episodes were asked to respond based on the experience they considered most relevant.

### Measures

Participants completed a survey consisting of five questionnaires, which they answered based on what they felt during the period in which they had DPDR-like experience(s), disregarding whether they had encountered similar experiences in other contexts. A sample survey is available on OSF (https://doi.org/10.17605/OSF.IO/YDQV4).

i. Cambridge depersonalisation scale (CDS)^21^. This 29-item questionnaire assesses various experiences of DPDR in terms of frequency (0 = *Never* to 4 = *All the time*) and duration (0 = *Never* to 6 = *More than a week*).
ii. Mysticism scale (MS). We used the 8-item short version^23^ of the MS^56^, which captures aspects such as unity, transcendence, and ineffability, with responses ranging from 0 (*Definitely not true*) to 4 (*Definitely true*).
iii. Ego dissolution inventory (EDI)^24^. This 8-item questionnaire evaluates various aspects of ego dissolution, such as dissolution of self, ego, or identity, and feelings of union with others or the universe. It is rated from 0 (*Not at all*) to 4 (*Extremely strongly*) and was originally developed to examine psychedelic experiences.
iv. Challenging experience questionnaire (CEQ)^22^. This 26-item tool examines various distressing emotions, such as sadness, fear, or despair, rated from 0 (*Not at all*) to 5 (*Extremely strongly*) and was originally developed to examine psychedelic experiences.
v. Five facet mindfulness questionnaire (FFMQ)^25^. Two of the five facets, the most relevant to the questions at hand, were used: (i) non-judging of inner experience (NJ; 8 items) and (ii) non-reactivity to inner experience (NR; 7 items). Items are rated on a scale from 1 (*Never or very rarely true*) to 5 (*Very often or always true*).

CDS scores were calculated by summing the items, whereas all other questionnaires were scored by averaging their items (with two separate scores derived for the FFMQ). For the CDS, MS, and EDI, participants also rated, if applicable, the emotional valence of each item on a 5-point scale (1 = *very negative* to 5 = *very positive*). An additional option, “negative & positive,” was available and coded as 3 (i.e., equivalent to “neutral”).

Participants were also asked whether their experiences resembled depersonalization, derealization, or both, and to rate the overall emotional valence of these states on a 7-point scale (1 = *very negative/unpleasant* to 7 = *very positive/pleasant*). They could select multiple valences (e.g., both positive and negative). They also provided demographic and clinical information, along with details about their meditation practice, if applicable.

### Data analysis

To assess demographic, clinical, and meditation variables, chi-square or permutation tests were used as appropriate. Participants with missing data for a particular measure were excluded from the corresponding analyses.

As preregistered, a MANCOVA was used to examine the effect of trigger (meditation vs. non-meditation) on questionnaire scores, adjusting for gender (with “diverse” grouped with “female”; see Supplementary Material for rationale), age, and mental health diagnosis (which was not part of the pre-registration, but which exploratory analyses revealed was different between groups). No multivariate outliers were found using Mahalanobis distance with a chi-square cutoff at *p* = 0.999 and df = 6 (reflecting six scores of interest). No multicollinearity or non-linear relationships were detected. One univariate outlier (|z|> 3) was found for the NR questionnaire, and analyses were repeated without it. To address assumption violations, permutation tests were conducted for each questionnaire. Deviations from the preregistered analysis plan were only minor and are described in the Supplementary Material. Additionally, since a CDS score ≥ 70 has been suggested as a clinical cutoff^21^, the main analysis was repeated for the subset of participants fulfilling this criterion (MEDT: *n* = 37, NMEDT: *n* = 47). Permutation tests were also run to compare groups on the five CDS factors^26^, overall emotional valence ratings, and emotional valence ratings of CDS, MS, and EDI items.

Chi-square tests were run to compare the proportion of participants in each group who selected a specific emotional valence of the DPDR-like experiences.

As preregistered, Pearson’s correlations were computed between all questionnaire scores. Additionally, correlations were assessed between: positive and negative emotional valence of DPDR experiences and questionnaire scores (see Supplementary Material for details on how valence was computed), and between questionnaire scores and the emotional valence of their respective items.

## Supplementary Information

Below is the link to the electronic supplementary material.

Supplementary Material 1

## Data Availability

Anonymized data and analysis code are available on OSF (https://doi.org/10.17605/OSF.IO/YDQV4).
