# Supplementary material for: A cross-sectional survey on depersonalization/derealization and meditation-induced alterations of the self
Source: Sci Rep. 2026 May 8;16:14673. doi: 10.1038/s41598-026-51014-y (PMC13156312; doi:10.1038/s41598-026-51014-y)
Supplement: Supplementary file 1 — Supplementary Material 1 [file 41598_2026_51014_MOESM1_ESM.pdf]

SUPPLEMENTARY MATERIAL FOR

A Cross-Sectional Survey on Depersonalization/Derealization and Meditation-Induced Alterations of the Self

Erola Pons[1,*], Julieta Galante[2], Nicholas T. Van Dam[2], Axel Lindner[1,3]

[1] Tübingen Center for Mental Health, Department of Psychiatry and Psychotherapy, University Hospital of Tübingen, Germany

[2] Contemplative Studies Centre, Melbourne School of Psychological Sciences, University of Melbourne, Australia

[3] Center of Neurology, Division of Neuropsychology, Hertie Institute for Clinical Brain Research, University of Tübingen, Tübingen, Germany

[*] Corresponding author: Erola Pons: erola.pw@gmail.com

Supplementary Methods: Data Analysis

Deviations from the preregistered analysis plan:

- Education was not included as a covariate in the MANCOVA as initially preregistered. This decision was made due to the large proportion of undergraduate students in the NMEDT group, who would have been categorized as "Not completed university degree." We determined that it was not meaningful to separate these participants from those who had already obtained a degree.
- A binary variable indicating the presence of a mental health diagnosis was included as a covariate in the MANCOVA (not specified in the preregistration) after a significant between-group difference in its prevalence was observed. The degrees of freedom (df) for the chi-square test used to identify multivariate outliers via Mahalanobis distance were set to 6, corresponding to the six scores of interest. This replaced the df = 5 specified in the preregistration, which was a mistake.

As stated in the Data Analysis section, we grouped “diverse” with “female” for the gender covariate in the MANCOVA. Although three response options were allowed, we aimed to analyze only two groups (female and male), and very few participants selected “diverse.” We grouped “diverse” with “female” rather than with “male” based on the generally more similar societal treatment of individuals identifying as diverse or female compared to male, and it also aligns with standard practice in the field.

Effect of trigger on questionnaire scores

Table S1. Effects of meditation (MEDT) and non-meditation (NMEDT) triggers on questionnaire scores (permutation tests).

	**MEDT (*n* = 60) M (*SD*)**	**NMEDT (*n* = 61) M (*SD*)**	***p***	$p_{corr.}$	**Cohen's *d***
Cambridge Depers. Scale (CDS)	92.83 (49.5)	108.54 (53.4)	0.100	0.598	-0.30
Mysticism Scale (MS)	3.11 (0.9)	1.59 (0.9)	<.001	<0.001*	1.69
Ego Dissolution Inventory (EDI)	2.79 (1.13)	1.36 (0.81)	<.001	<0.001*	1.44
Challenging Exper. Quest. (CEQ)	1.98 (1.48)	2.64 (1.18)	0.007	0.040*	-0.50
Non-Judging (NJ)	3.09 (0.74)	1.81 (1.12)	<.001	<0.001*	1.34
Non-Reactivity (NR)	2.87 (0.72)	1.47 (0.83)	<.001	<0.001*	1.81

Subset of high-DPDR participants (CDS score ≥70)

37 participants in the MEDT group (61.7%) and 47 in the NMEDT group (77.9%) had a CDS score of 70 or above, considered to be a clinically relevant DPDR. We repeated the main analysis for this subset of participants. A one-way MANCOVA revealed a significant multivariate effect of trigger on the combined questionnaire scores after controlling for gender and age, Pillai's trace = 0.658, $F(6,75)=24.058$, $p<.001$, partial $\eta^2 = .397$. The effects of gender and age were non-significant ($p>.05$). Subsequent permutation tests on the individual questionnaire scores revealed a significant impact of trigger on all questionnaires except the CDS and the CEQ, after Bonferroni correction (Table S1).

Table S2. Effects of meditation (MEDT) and non-meditation (NMEDT) triggers on questionnaire scores (permutation tests).

	**MEDT (*n* = 37) M (*SD*)**	**NMEDT (*n* = 47) M (*SD*)**	***p***	$p_{corr.}$	**Cohen's *d***
Cambridge Depers. Scale (CDS)	120.16 (43.97)	128.02 (43.99)	0.419	1.000	-0.18
Mysticism Scale (MS)	3.27 (0.84)	1.63 (0.88)	<.001	<0.001*	1.90
Ego Dissolution Inventory (EDI)	3.1 (1.06)	1.51 (0.78)	<.001	<0.001*	1.75
Challenging Exper. Quest. (CEQ)	2.39 (1.55)	2.88 (1.08)	0.094	0.566	-0.38
Non-Judging (NJ)	3.06 (0.81)	1.67 (1.15)	<.001	<0.001*	1.37
Non-Reactivity (NR)	1.09 (0.85)	2.51 (0.84)	<.001	<0.001*	1.68

Number of valences endorsed

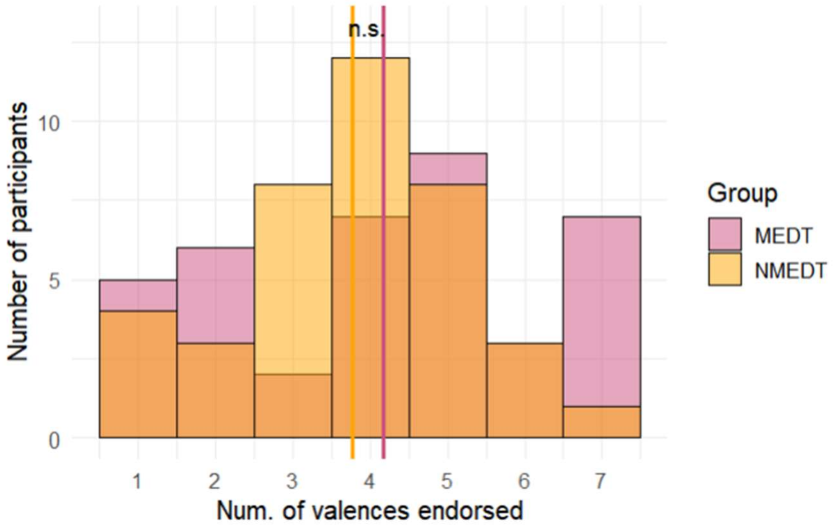

Figure S1. Number of valences endorsed by MEDT and NMEDT participants. Solid vertical lines indicate group means; the difference is not significant ("n.s.", permutation test).

Emotional valence of the DPDR-like experiences

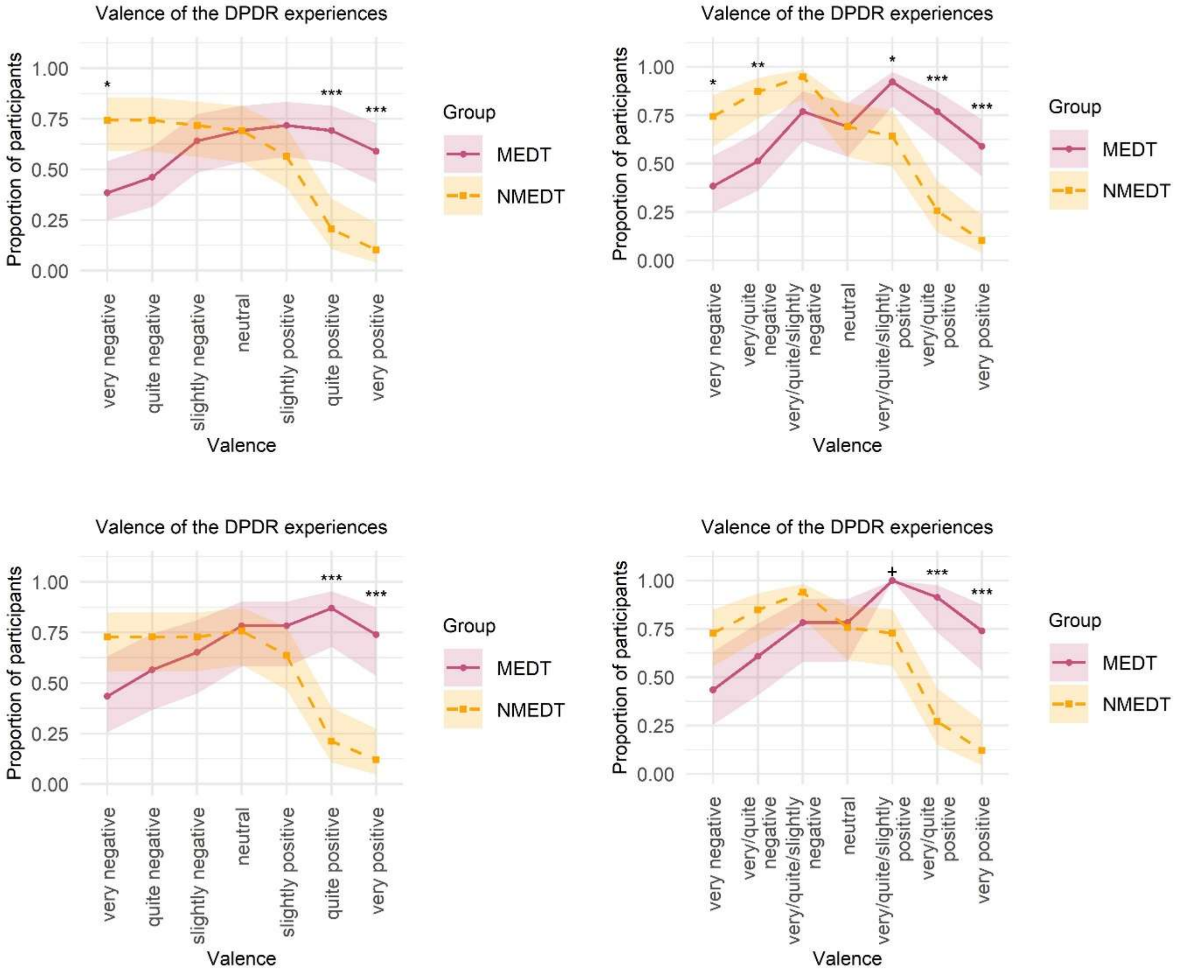

Figure S2. Emotional valence of the DPDR-like experiences for the two groups. Top plots show data for all participants; bottom plots include only those with CDS ≥ 70. Left panels display raw response proportions (e.g., % selecting "very negative"); right panels show cumulative proportions (e.g., % selecting "very negative" or "quite negative" or "slightly negative"). Shaded areas = 95% CIs. Significance symbols (+ < .1, * < .05, ** < .01, *** < .001) reflect Bonferroni-corrected chi-square tests.

Valence of the questionnaire items (for CDS, MS, and EDI)

For the CDS, MS, and EDI, participants rated the emotional valence of each item on a 5-point scale (1 = *very negative* to 5 = *very positive*). An additional option, "negative & positive," was available and coded as 3 (i.e., equivalent to "neutral").

Table S3. Effects of meditation (MEDT) and non-meditation (NMEDT) triggers on questionnaire valence (permutation tests).

	**MEDT (n = 46)** **M (*SD*)**	**NMEDT (n = 38)** **M (*SD*)**	p	$p_{corr.}$	**Cohen's *d***
**Cambridge Depers. Scale (CDS)**	3.40 (0.73)	2.15 (0.60)	<.001	<.001	1.85
**Mysticism Scale (MS)**	4.35 (0.61)	2.81 (0.87)	<.001	<.001	2.09
**Ego Dissolution Inventory (EDI)**	4.25 (0.67)	2.50 (0.85)	<.001	<.001	2.32

DPDR phenomenology

Table S4. Differences between the triggers meditation and non-meditation on the CDS factors. Mean scores (SD), t statistic, p-values (corrected), and effect size (Cohen's *d*) are represented.

	MEDT (*n* = 46) M (*SD*)	NMEDT (*n* = 38) M (*SD*)	*p*	$p_{corr.}$	Cohen's *d*
**Numbing**	14.67 (13.09)	19.46 (14.46)	0.061	0.306	-0.35
**Unreality of Self**	24.95 (13.67)	27.84 (13.63)	0.257	1.000	-0.21
**Perceptual Alterations**	11.00 (8.71)	11.67 (9.38)	0.691	1.000	-0.07
**Unreality of Surroundings**	7.58 (5.08)	10.10 (5.55)	0.010	0.050	-0.47
**Temporal Disintegration**	11.90 (7.88)	15.15 (9.42)	0.043	0.214	-0.37

Table S5. Top items of the CDS endorsed by each of the groups.

	**MEDT**
**CDS06**	Whilst doing something I have/had the feeling of being a 'detached observer' of myself.
**CDS26**	I feel/felt so detached from my thoughts that they seem/seemed to have a 'life' of their own.
**CDS04**	I have/had found myself not being frightened at all in situations which normally I would find frightening or distressing.
**CDS01**	Out of the blue, I feel/felt strange, as if I were not real or as if I were cut off from the world.
**CDS10**	I have/had the feeling of not having any thoughts at all, so that when I speak/spoke it feels/felt as if my words were being uttered by an 'automaton'.
	**NMEDT**
**CDS13**	My surroundings feel/felt detached or unreal, as if there were a veil between me and the outside world.
**CDS01**	Out of the blue, I feel/felt strange, as if I were not real or as if I were cut off from the world.
**CDS06**	Whilst doing something I have/had the feeling of being a 'detached observer' of myself.
**CDS05**	My favourite activities are/were no longer enjoyable.
**CDS16**	I feel/felt detached from memories of things that have/had happened to me – as if I had not been involved in them.

Other meditation types reported (MEDT)

Table S6. Open-Text “Other” Meditation Practices Reported as Preceding DPDR in the MEDT group. Some responses are abridged for anonymity.

**Led to DPDR**
Mixture of Christo-Judeo + Buddhism + alchemy […] one technique is called “the bottomless pit”/“crossing the abyss” (transcending the mind). There's a slight emphasis on the sense of self.
Other FA (e.g., vast space, silence)
Pratyahara
Emptiness Meditations
Homebrew noting with extreme emphasis on cause and effect through the entire typical insight cycle
Kasina
Psychotherapy: Psychodynamic therapy, Shadow work (like Internal Family Systems), EMDR
Somatic Compassion Practices, Guru Yoga
Focusing on the beauty of water during showers
Progressive muscle relaxation, guided “hypnosis” (app)
"Tratak" form of meditation which involves staring at external/internal objects
the buddhist way of Dhammakaya
For instance looking at my body parts with love and care it deserved. […]
TMI Style Samatha and Samatha-Vipassana
Osho Meditations, Tantra
[…] "soft" jhana, as i would focus on pure consciousness, pure spaciousness, emptiness, etc. and the other kind of vipassana i did was a self-guided undoing or dissolving of all mental fabrications of "me" and also the reality of the objects […]
Analytical / Buddhist Vipassana
Jungian active imagination
Focus on emotions in the body
Unguided meditation - focusing on ambient sounds in the environment
Fire Kasina
Guided meditations/visualisations
Nada Sound Practice & Ashtanga Yoga
**Did not lead to DPDR**
Reading books mostly from Theravada, as well as Hussel and other moder phenomenologists (that also had an influence in him as well)
shamanic journeying, dream yoga (working with dreams), energy work, imaginal practice (Jung)
trataka
MBSR (including eating and walking meditation)
[…] sit in my room on the cold floor and be aware of the sounds, the surroundings heat sensations and my heart beat […]<br>Visualizations
Kriya Yoga is basically kundalini stuff but we use pranayama and a mix of other techniques using the om mantra in various specific ways to accomplish various psycho-emotional goals and states of Samadhi.
Tonglen / Visualisation
Insight Timer guided meditations
Fire Kasina, Vizualizations
Contemplation of the six elements
Kiloby Inquiry meditation

DPDR experiences due to meditation *and* other triggers (MEDT)

Table S7. Permutation test results within the MEDT group comparing those participants who had also experienced a DPDR-like experience due to other triggers (e.g., cannabis, depression, stress, anxiety, trauma) vs. those who had not. n = 60 for the questionnaires; n = 39 for the valences.

	**OtherTrigger = 1 (n = 30) M (*SD*)**	**OtherTrigger = 0 (n = 30) M (*SD*)**	p	$p_{corr.}$	**Cohen's *d***
Cambridge Depers. Scale (CDS)	92.47 (54.41)	93.20 (44.99)	0.958	1.000	-0.01
Mysticism Scale (MS)	3.15 (0.81)	3.08 (0.99)	0.793	1.000	0.07
Ego Dissolution Inventory (EDI)	2.75 (1.14)	2.83 (1.15)	0.814	1.000	-0.07
Challenging Exper. Quest. (CEQ)	2.05 (1.50)	1.90 (1.49)	0.698	1.000	0.10
Non-Judging (NJ)	2.88 (0.82)	3.29 (0.60)	0.036	0.281	-0.56
Non-Reactivity (NR)	2.88 (0.62)	2.87 (0.81)	1.000	1.000	0.01
Negative Valence	1.95 (1.15)	1.37 (1.26)	0.152	1.000	0.48
Positive Valence	2.45 (0.83)	2.11 (1.15)	0.337	1.000	0.35

Table S8. Permutation test results within the MEDT group comparing those participants who had also experienced a DPDR-like experience due to psychedelics vs. those who had not. n = 60 for the questionnaires; n = 39 for the valences.

	**Psychedelics = 1 (n = 25) M (*SD*)**	**Psychedelics = 0 (n = 35) M (*SD*)**	p	$p_{corr.}$	**Cohen's *d***
Cambridge Depers. Scale (CDS)	92.24 (54.57)	93.26 (46.36)	0.942	1.000	-0.02
Mysticism Scale (MS)	3.33 (0.69)	2.95 (1.00)	0.106	0.775	0.43
Ego Dissolution Inventory (EDI)	2.90 (1.03)	2.71 (1.21)	0.530	1.000	0.17
Challenging Exper. Quest. (CEQ)	2.21 (1.55)	1.81 (1.43)	0.310	1.000	0.27
Non-Judging (NJ)	3.10 (0.83)	3.08 (0.69)	0.950	1.000	0.02
Non-Reactivity (NR)	2.95 (0.59)	2.82 (0.79)	0.475	1.000	0.19
Negative Valence	2.00 (1.12)	1.41 (1.26)	0.146	1.000	0.49
Positive Valence	2.47 (0.94)	2.14 (1.04)	0.346	1.000	0.34
